# A case–control study investigating the impact of gradient pressure therapy on chemotherapy-induced peripheral neuropathy symptoms and daily activities in paclitaxel-treated patients

**DOI:** 10.1186/s43046-025-00331-w

**Published:** 2025-11-24

**Authors:** Dongxue Guo, Fangfei Zhao, JiWei Hu, JingHua Zhang, Aijun Du, Lizhi Zhou

**Affiliations:** 1 North China University of Science and Technology, Tangshan, China; 2 North China University of Science and Technology Affiliated Hospital, Tangshan, China; 3 Tangshan People’s Hospital, Tangshan, China; 4 Tangshan Maternal and Child Health Hospital, Tangshan, China

**Keywords:** Peripheral neuropathy, Breast cancer, Chemotherapy, Pressure therapy, Paclitaxel, Quality of life

## Abstract

**Purpose:**

Paclitaxel chemotherapy is known to induce peripheral neuropathy, significantly impacting patients' daily activities. Effective interventions to alleviate these symptoms are crucial for improving patient quality of life. The objective was to investigate the effects of gradient pressure therapy on symptoms of paclitaxel-induced peripheral neuropathy and daily activities.

**Methods:**

Eighty patients with breast cancer receiving a 125-mg/m^2^ paclitaxel chemotherapy regimen at a class III hospital in Tangshan from October 2022 to October 2023 were randomly divided into a control group (*n* = 40) and an intervention group (*n* = 40). The control group received routine treatment and health guidance, whereas the intervention group received additional II-level hand and foot gradient pressure therapy. Chemotherapy-induced peripheral neuropathy (CIPN) symptoms and activities of daily living (ADL) impact scores were compared after the second, fourth, and sixth chemotherapy cycles and at the 3-month follow-up.

**Results:**

No significant differences were found in CIPN symptoms and ADL impact scores between the groups after the second chemotherapy cycle (*P* > 0.05). However, after the fourth cycle, the intervention group showed significant improvements in nine items: hand numbness, foot numbness, discomfort in fingers/hands or toes/feet, walking, exercise, sleep, sexual activity, chores, and enjoyment of life (*P* < 0.05). At the sixth cycle and at the 3-month follow-up, additional improvements were noted in cold sensitivity and total scores (*P* < 0.05).

**Conclusion:**

Gradient pressure therapy effectively reduces symptoms of paclitaxel-induced peripheral neuropathy, improving patients’ daily activities. This intervention is recommended for clinical promotion and application.

## Introduction

Breast cancer is the most common malignant tumor in women, posing a threat to their physical and mental health [1]. Currently, chemotherapy is the primary treatment; however, it has various side effects [2]. The most common side effect is chemotherapy-induced peripheral neuropathy (CIPN), mainly caused by paclitaxel and platinum-containing drugs [3]. CIPN incidence induced by these drugs is > 70% [4]. CIPN is a dose-limiting adverse reaction that can damage sensory, motor, and autonomic nerves, manifesting as bilateral limb numbness, tingling, muscle weakness, balance disorders, and difficulty performing fine movements. These symptoms can persist for several months or years post-chemotherapy [5]. Intolerable symptoms may lead some patients to reduce drug doses or discontinue treatment, resulting in poor treatment outcomes and prognosis, ultimately affecting their daily activities and quality of life [6, 7]. Studies from countries outside China [8–10] have confirmed that compression therapy can effectively prevent or reduce CIPN incidence. However, they often use self-controlled trials without specifying pressure values and durations, lacking detailed analyses of CIPN symptoms and their impact on activities of daily living (ADL). This area receives limited attention in China. Therefore, this study aimed to explore the intervention effects of gradient pressure therapy on CIPN symptoms and ADL induced by albumin-paclitaxel.

## Methods

### Study design and patients

Eighty patients with breast cancer undergoing albumin-paclitaxel chemotherapy in the breast surgery department of a class III grade A hospital in Tangshan between October 2022 and October 2023 were selected as research participants. The sample size was calculated using the formula for randomized controlled trial (two-group rate comparison): *n* = $\frac{2\overline{p }\overline{q }{({Z}_{\alpha }+{Z}_{\beta })}^{2}}{{(p1-p2)}^{2}}$. Here, n represents the sample size of each group, α = 0.05, taking both sides, β = 0.1, according to the table, Z_*α*_ = 1.96, Z_*β*_ = 1.28, p1 and p2 represent the incidence of the control group and the intervention group, respectively, $\overline{p }$ represents the mean of p1 and p2, and $\overline{q }$ represents the mean of 1-p1 and 1-p2. Based on literature reviews, a sample size of *n* = 36 was calculated and increased by 10% to account for potential losses, resulting in *n* = 40 and a final total sample size of 80 cases. The RAND function in Excel was used for randomization, selecting 80 random numbers and placing them in sealed envelopes. After the patients agreed to participate in the study, a data set was randomly selected; odd numbers were assigned to the control group and even numbers to the intervention group, with 40 cases in each group.

This study was reviewed and approved by the Ethics Committee of Tangshan People’s Hospital (RMYY-LLKS-2022–054), and informed consent was obtained from all participants.

### Inclusion and exclusion criteria

Inclusion criteria comprised: (1) breast cancer diagnosed by pathological examination; age ≥ 18 years; (2) first chemotherapy with albumin-paclitaxel at 125 mg/m^2^ every 21 days for 6 cycles; (3) ability to listen, read, write, and complete the questionnaire independently; and (4) ability to provide informed consent.

Exclusion criteria were (1) previous central nervous system or mental diseases; (2) rheumatoid arthritis; (3) interruption, adjustment, or withdrawal of the chemotherapy regimen; (4) severe organ dysfunction; and (5) allergy to chemotherapy drugs and latex materials.

### Treatments and compressive therapy intervention

Control group: routine treatment and health guidance were provided, including (1) pre-chemotherapy education on precautions, adverse reactions, and preventive measures; (2) dietary guidance to maintain a light, easy-to-digest, and balanced diet including meat and vegetables; and (3) others: advice on rest, work-life balance, and regular monitoring of parameters including blood routine and liver and kidney function.

Intervention group:

1) Preliminary experiment: participants meeting the inclusion criteria were selected for the preliminary experiment. Literature reviews, including studies by Tsuyuki et al. [8, 9], showed that the use of surgical gloves to compress the whole hand, including the fingers, could effectively reduce the incidence of sensory and motor peripheral neuropathy of the hand without observations of patient intolerance or adverse events such as skin allergies or pruritus. Wu et al. [11] showed that compression therapy using surgical gloves one size smaller than the appropriate size and grade II high-pressure long elastic socks could reduce the occurrence of acute oxaliplatin-induced peripheral neuropathy without adverse reactions. Based on the study design of relevant literature on compression therapy, the intervention duration was determined to be 90 min: 30 min before chemotherapy drug infusion, 30 min after chemotherapy drug infusion, and 30 min to control the infusion time of albumin-paclitaxel.

The pressure classification of medical stretch socks is divided into four grades according to the European standard. According to their length, they are divided into short socks, long socks, and pantyhose. Based on the design of the toe end, they are classified into closed-toe and open-toe styles. Because CIPN symptoms often occur in both hands and feet, all patients were given surgical gloves and short-cuffed closed-toe medical elastic stockings. The patients were instructed to wear smaller surgical gloves and different pressure-graded compression stockings according to the research protocol, and their tolerance, comfort, and adverse reactions were observed. Preliminary experimental results showed no patient withdrawals and no adverse reactions. However, Yamanouchi et al. (2022) found that 15–20 mmHg did not prevent docetaxel-induced peripheral neuropathy and that patients who wore level III or higher compression stockings felt uncomfortable [12]. Combined with the study by Liling W et al. (2021), level II pressure was chosen for the intervention [11].

2) Final research proposal: based on the control group, the patients received grade II pressure (23–32 mmHg) gradient compression therapy. This involved wearing surgical gloves smaller than their suitable size and grade II pressure toe elastic socks. This process was as follows: (1) the palm width of the patients in the intervention group was measured using a soft ruler from the midpoint of the roots of the index finger and thumb to the edge of the palm (https://www.chinesestandard.net/Related.aspx/YYT0851-2023) [13]. According to the measured results and the size chart of the surgical gloves, smaller surgical gloves were selected for the patients. Subsequently, the patients stood while the smallest part of their ankle circumference and the largest part of their calf circumference were measured using a soft ruler (https://www.chinesestandard.net/PDF/English.aspx/GBT7543-2020) [14]. Based on these measurements and the medical elastic sock model, the appropriate size of grade II pressure short-toe elastic socks was selected. (2) Patients were instructed to wear surgical gloves and medical elastic socks. Using a pressure-measuring instrument, we ensured the pressure on their fingers, toes, feet, and heels reached grade II pressure. If the pressure did not fall within the grade II range, another pair of surgical gloves or cotton socks were added. The pressure values showed a gradual decrease from the distal to the proximal end, forming a pressure gradient that promoted rapid blood flow. (3) Intervention time: Patients wore gloves for 90 min continuously, starting 30 min before and 30 min after the infusion of albumin-paclitaxel in each cycle. The researcher measured patients’ pressure points at each admission. (4) Safety assessment: we monitored the tolerability of pressure therapy and any adverse reactions related to the use of surgical gloves and medical compression stockings. Any abnormalities, such as allergies, rashes, and skin itching, were actively managed and documented. Circulation in the extremities was also monitored, and symptoms such as purple discoloration, swelling, or pain were promptly treated.

### Evaluation of peripheral neuropathy

The Chemotherapy-Induced Peripheral Neuropathy Assessment Tool (CIPNAT) was used to evaluate patients in both groups after the second, fourth, and sixth cycles of chemotherapy and at the 3-month follow-up. This tool was developed by Tofthagen et al. in the USA [15] and translated into Chinese by Wang et al. (2018) [16]. In this study, the Chinese version of the tool with Cronbach's α coefficients ranging from 0.89 to 0.94 was used for the total scale and each subscale.

The CIPNAT tool consists of two parts. The first part assesses the experience of CIPN symptoms, which includes nine symptoms and ranges from 0 to 279 points, with higher scores indicating worse CIPN symptom experience. The second part evaluates the impact of CIPN on ADL with 14 items, with scores ranging from 0 to 10, for a total score range of 0–140 points. Higher scores indicate a greater impact of CIPN symptoms on the patient’s ADL.

### Statistical analysis

Statistical analyses were performed using SPSS 26.0. Continuous data were described using mean ± standard deviation (x ® ± s), and group comparisons were made using independent sample *t-*tests. Non-normal data were described using median [M (P25, P75)], and group comparisons were performed using the rank-sum test. Count data were described using frequencies and percentages, with group comparisons made using the chi-square test. Categorical data were represented by the number of cases, and group comparisons were performed using non-parametric tests. *P* < 0.05 was considered statistically significant.

## Results

### Patient general characteristics

There was no significant difference in the general information between the two groups of patients, including age, body mass index, education level, medical payment, family/personal monthly income, tumor stage, surgery, medical history, and family history of cancer (*P* > 0.05) (Table 1).

**Table 1:** General patient characteristics (*n*, %)

Characteristic	Sort	Control (*n* = 40)	Intervention (*n* = 40)	*t*/χ^2^/*Z*	*P*
Age, years ($\overline{x }\pm s)$	—	51.40 ± 10.42	51.68 ± 11.00	0.115^a^	0.909
BMI (kg/m^2^)	—	25.30 ± 3.11	25.31 ± 3.79	0.017^a^	0.986
Education level	Primary school and below	10 (25.0)	10 (25.0)	− 0.604^c^	0.546
	Junior high school	18 (45.0)	13 (32.5)		
	High school or technical secondary school	6 (15.0)	11 (27.0)		
	Junior college and undergraduate	6 (15.0)	6 (15.0)		
Medical payment	Employee medical insurance	11 (27.5)	8 (20.0)	0.621^b^	0.431
	Urban medical insurance	29 (72.5)	32 (80.0)		
Household/per capita monthly (income, CNY)	< 1000	13 (32.5)	17 (42.5)	− 0.321^c^	0.748
	1000–3000	12 (30.0)	7 (17.5)		
	3000–5000	9 (22.5)	9 (22.50)		
	> 5000	6 (15.0)	7 (17.50)		
Tumor stage	I	7 (17.5)	8 (20.0)	− 0.178^c^	0.858
	II	28 (70.0)	25 (62.5)		
	III	5 (12.5)	7 (17.5)		
Surgery	Yes	15 (37.5)	16 (40.0)	0.053^b^	0.818
	No	25 (62.5)	24 (60.0)		
Previous history	Hypertension	6 (15.0)	10 (25.0)	1.250^b^	0.264
	No	34 (85.0)	30 (75.0)		
Family history of cancer	Yes	9 (22.5)	7 (17.5)	0.313^b^	0.576
	No	31 (77.5)	33 (82.5)		

^a^*t *value

^b^χ^2^

^c^*Z* value

### Comparison of CIPNAT scores between the two groups of patients after the second, fourth, and sixth chemotherapy cycles and at the 3-month follow-up

1) Comparison of CIPN symptoms experienced by patients in both groups after the second, fourth, and sixth chemotherapy cycles and at the 3-month follow-up.

There was no significant difference in the scores of each item and the total score of CIPN symptoms experienced by patients in both groups after the second chemotherapy cycle (*P* > 0.05). There were significant differences in the scores for numbness in the hands, numbness in the feet, uncomfortable fingers/hands or toes/feet, and total points between the two groups after the fourth chemotherapy cycle (*P* < 0.05); however, no significant differences were observed for other items (*P* > 0.05). There were significant differences in the scores for numbness in the hands, numbness in the feet, uncomfortable fingers/hands or toes/feet, cold sensitivity, and total point between the two groups after the sixth chemotherapy cycle and at the 3-month follow-up (*P* < 0.05); other items showed no significant differences (*P* > 0.05). No patients in either group experienced tingling in the hands and feet during chemotherapy. During the treatment period, the total score of CIPN symptom experience showed different degrees of increase over time. The trend in the intervention group was relatively slow and showed a downward trend after chemotherapy (Table 2).

**Table 2:** Comparison of CIPN symptom experience items and total score in the two groups of patients after the second, fourth, and sixth chemotherapy cycles and at the 3-month follow-up [M (P_25_, P_75_), score]

Item		Time node	Control (*n* = 40)	Intervention (*n* = 40)	*Z*	*P*
Numbness in the hands	Cycle 2		0.00 (0.00, 12.00)	0.00 (0.00, 10.75)	− 0.899	0.368
	Cycle 4		15.00 (13.00, 16.75)	10.50 (0.00, 14.00)	− 4.138	< 0.001
	Cycle 6		18.50 (16.25, 19.00)	15.00 (0.00, 16.75)	− 5.224	< 0.001
	Follow-up for 3 months		0.00 (0.00, 14.5)	0.00 (0.00, 0.00)	− 2.103	0.035
Numbness in the feet	Cycle 2		6.50 (0.00, 12.00)	0.00 (0.00, 11.00)	− 1.563	0.118
	Cycle 4		16.00 (14.00, 19.00)	13.00 (0.00, 15.00)	− 3.710	< 0.001
	Cycle 6		18.00 (16.00, 20.00)	16.00 (3.00, 16.00)	− 4.687	< 0.001
	Follow-up for 3 months		0.00 (0.00, 17.00)	0.00 (0.00, 10.00)	− 2.063	0.039
Uncomfortable fingers/hands or toes/feet	Cycle 2		11.50 (0.00, 15.00)	10.00 (0.00, 13.00)	− 1.395	0.163
	Cycle 4		14.00 (12.00, 15.00)	17.00 (15.00, 19.00)	− 4.636	< 0.001
	Cycle 6		19.00 (17.25, 22.00)	15.00 (14.00, 17.75)	− 5.365	< 0.001
	Follow-up for 3 months		4.50 (0.00, 15.75)	0.00 (0.00, 13.75)	− 2.058	0.040
Cold sensitivity	Cycle 2		0.00 (0.00, 3.75)	0.00 (0.00, 0.00)	− 0.915	0.360
	Cycle 4		7.00 (0.00, 10.00)	0.00 (0.00, 10.00)	− 1.104	0.270
	Cycle 6		12.00 (0.00, 13.00)	7.00 (0.00, 11.00)	− 3.248	0.001
	Follow-up for 3 months		0.00 (0.00, 0.00)	0.00 (0.00, 0.00)	− 2.355	0.019
Muscle weakness	Cycle 2		0.00 (0.00, 5.75)	0.00 (0.00, 0.00)	− 0.813	0.416
	Cycle 4		8.00 (0.00, 11.00)	0.00 (0.00, 9.75)	− 1.235	0.217
	Cycle 6		11.00 (0.00, 13.75)	4.50 (0.00, 11.75)	− 1.826	0.068
	Follow-up for 3 months		0.00	0.00	—	—
Weakness in the arms or legs	Cycle 2		0.00 (0.00, 12.00)	0.00 (0.00, 9.00)	− 0.707	0.480
	Cycle 4		10.50 (0.00, 13.75)	13.00 (0.00, 15.00)	− 1.161	0.246
	Cycle 6		13.50 (11.25, 17.00)	12.50 (0.00, 16.00)	− 1.464	0.143
	Follow-up for 3 months		0.00 (0.00, 9.00)	0.00 (0.00, 5.25)	− 0.554	0.580
Loss of balance	Cycle 2		0.00	0.00	—	—
	Cycle 4		0.00 (0.00, 8.00)	0.00 (0.00, 0.00)	− 1.291	0.197
	Cycle 6		7.00 (0.00, 11.00)	0.00 (0.00, 9.75)	− 1.293	0.196
	Follow-up for 3 months		0.00	0.00	—	—
Total points	Cycle 2		31.50 (0.00, 48.00)	25.00 (0.00, 42.50)	− 1.309	0.191
	Cycle 4		76.50 (59.00, 87.00)	51.00 (28.50, 69.50)	− 3.526	< 0.001
	Cycle 6		98.50 (76.00, 106.00)	70.00 (44.75, 82.50)	− 4.392	< 0.001
	Follow-up for 3 months		16.50 (0.00, 51.75)	0.00 (0.00, 38.25)	− 1.988	0.047

2) Comparison of the effects of CIPN on ADL in the two groups of patients after the second, fourth, and sixth chemotherapy cycles and at the 3-month follow-up.

There was no significant difference in the scores of each item and the total score of CIPN on ADL between the two groups of patients after the second cycle of chemotherapy (*P* > 0.05). Significant differences were noted in the scores for walking, exercise, sleep, sexual activity, chores, and enjoyment of life between the two groups of patients after the fourth and sixth cycles of chemotherapy and at the 3-month follow-up (*P* < 0.05); other items showed no significant differences (*P* > 0.05). The total CIPN score on ADL increased at different rates over time during the treatment period, with the intervention group showing a relatively slow increase and a downward trend after chemotherapy (Table 3).

**Table 3:** Comparison of CIPN effects on ADL in the two groups of patients after the second, fourth, and sixth cycles of chemotherapy and at the 3-month follow-up [M (P25, P75), score]

Item	Time node	Control (*n* = 40)	Intervention (*n* = 40)	*Z*	*P*
Dressing	Cycle 2	0.50 (0.00, 1.75)	0.00 (0.00, 1.00)	− 1.100	0.271
	Cycle 4	2.00 (2.00, 3.00)	2.00 (1.00, 2.00)	− 1.367	0.172
	Cycle 6	2.00 (2.00, 3.00)	2.00 (1.00, 3.00)	− 1.459	0.144
	Follow-up for 3 months	0.00 (0.00, 1.00)	0.00(0.00, 0.75)	− 1.469	0.142
Walking	Cycle 2	1.00 (0.00, 1.75)	0.50 (0.00, 2.00)	− 0.472	0.637
	Cycle 4	3.00 (2.00, 4.00)	2.00 (1.00, 2.00)	− 2.810	0.005
	Cycle 6	4.00 (3.00, 5.00)	3.00 (2.00, 4.00)	− 3.341	0.001
	Follow-up for 3 months	2.00 (0.00, 3.00)	0.00 (0.00, 2.00)	− 2.080	0.037
Picking up objects	Cycle 2	0.00	0.00	—	—
	Cycle 4	1.00 (0.00, 2.00)	1.00 (0.00, 1.00)	− 1.168	0.243
	Cycle 6	1.00 (1.00, 2.00)	1.00 (0.00, 2.00)	− 1.381	0.167
	Follow-up for 3 months	0.00 (0.00, 0.00)	0.00 (0.00, 0.00)	− 1.245	0.213
Holding onto objects	Cycle 2	0.00	0.00	—	—
	Cycle 4	1.00 (0.00, 2.00)	1.00 (0.00, 1.00)	− 1.169	0.242
	Cycle 6	1.00 (1.00, 2.00)	1.00 (0.00, 2.00)	− 1.205	0.228
	Follow-up for 3 months	0.00 (0.00, 0.75)	0.00 (0.00, 0.00)	− 1.781	0.075
Driving	Cycle 2	1.00 (0.00, 2.00)	0.00 (0.00, 1.00)	− 1.509	0.131
	Cycle 4	2.00 (1.00, 3.00)	2.00 (1.00, 2.00)	− 1.527	0.127
	Cycle 6	1.00 (0.00, 2.00)	1.00 (0.00, 1.00)	− 1.227	0.220
	Follow-up for 3 months	0.00 (0.00, 0.00)	0.00 (0.00, 0.00)	− 1.436	0.151
Working	Cycle 2	1.00 (0.00, 2.00)	1.00 (0.00, 2.00)	− 1.072	0.284
	Cycle 4	2.00 (1.00, 2.00)	1.50 (0.00, 2.00)	− 1.315	0.189
	Cycle 6	1.00 (0.00, 2.75)	0.00 (0.00, 2.00)	− 1.593	0.111
	Follow-up for 3 months	0.00 (0.00, 0.00)	0.00 (0.00, 0.00)	− 1.473	0.141
Hobbies	Cycle 2	2.00 (0.00, 3.00)	1.00 (0.00, 2.00)	− 1.598	0.110
	Cycle 4	2.00 (1.00, 2.00)	2.00 (0.00, 2.00)	− 1.538	0.124
	Cycle 6	2.00 (1.25, 2.00)	2.00 (1.00, 2.00)	− 1.603	0.109
	Follow-up for 3 months	0.00 (0.00, 1.75)	0.00 (0.00, 1.00)	− 1.092	0.275
Exercise	Cycle 2	2.00 (0.00, 2.00)	1.00 (0.00, 2.00)	− 1.382	0.167
	Cycle 4	2.50 (2.00, 3.00)	2.00 (1.00, 2.00)	− 2.222	0.026
	Cycle 6	5.00 (4.00, 5.00)	2.00 (0.25, 3.75)	− 4.985	< 0.001
	Follow-up for 3 months	1.00 (0.00, 3.00)	0.00 (0.00, 2.00)	− 2.166	0.030
Sleep	Cycle 2	1.00 (0.00, 2.00)	0.00 (0.00, 2.00)	− 1.391	0.164
	Cycle 4	3.00 (3.00, 5.00)	2.00 (2.00, 3.00)	− 3.125	0.002
	Cycle 6	5.00 (4.00, 6.00)	4.00 (2.00, 5.00)	− 4.266	< 0.001
	Follow-up for 3 months	2.00 (0.00, 3.00)	0.00 (0.00, 2.00)	− 2.218	0.027
Sexual activity	Cycle 2	2.00 (0.00, 2.00)	1.00 (0.00, 2.00)	− 1.474	0.141
	Cycle 4	2.00 (1.00, 3.00)	1.00 (1.00, 2.00)	− 3.507	< 0.001
	Cycle 6	3.00 (2.00, 4.00)	1.00 (0.25, 2.00)	− 4.167	< 0.001
	Follow-up for 3 months	0.00(0.00, 1.00)	0.00 (0.00, 0.00)	− 2.188	0.029
Relationship	Cycle 2	0.00	0.00	—	—
	Cycle 4	2.00 (2.00, 3.00)	2.00 (1.00, 2.75)	− 1.423	0.155
	Cycle 6	2.00 (1.25, 3.00)	2.00 (1.00, 2.00)	− 1.524	0.127
	Follow-up for 3 months	0.00 (0.00, 0.00)	0.00 (0.00, 0.00)	− 0.422	0.673
Writing	Cycle 2	0.00 (0.00, 0.00)	0.00 (0.00, 0.00)	− 1.154	0.249
	Cycle 4	2.00 (0.00, 2.00)	1.00 (0.25, 2.00)	− 1.108	0.268
	Cycle 6	1.00 (0.25, 2.00)	1.00 (1.00, 2.00)	− 1.088	0.277
	Follow-up for 3 months	0.00 (0.00, 0.00)	0.00 (0.00, 0.00)	− 1.079	0.280
Doing chores	Cycle 2	2.00 (0.00, 3.00)	1.00 (0.00, 2.00)	− 1.330	0.183
	Cycle 4	4.00 (2.00, 5.00)	3.00 (1.00, 3.00)	− 3.464	0.001
	Cycle 6	5.00 (4.00, 7.00)	4.00 (0.00, 5.00)	− 3.695	< 0.001
	Follow-up for 3 months	0.00 (0.00, 2.75)	0.00 (0.00, 1.50)	− 2.074	0.038
Enjoyment of life	Cycle 2	2.00 (0.00, 2.00)	0.00 (0.00, 2.00)	− 1.877	0.061
	Cycle 4	4.50 (4.00, 6.00)	4.00 (2.25, 4.75)	− 3.023	0.003
	Cycle 6	7.00 (6.00, 8.75)	5.00 (4.25, 6.00)	− 5.212	< 0.001
	Follow-up for 3 months	3.00 (0.00, 4.75)	0.00 (0.00, 3.00)	− 2.229	0.026
Total points	Cycle 2	16.00 (0.00, 20.75)	13.00 (0.00, 18.75)	− 1.837	0.066
	Cycle 4	35.00(28.00,40.00)	27.00(19.25, 31.00)	− 3.918	< 0.001
	Cycle 6	44.00(38.25,49.00)	32.00 (26.50, 36.00)	− 5.957	< 0.001
	Follow-up for 3 months	12.00 (0.00, 21.50)	0.00 (0.00, 14.50)	− 2.252	0.024

### Adverse events associated with gradient compression therapy

Gradient compression therapy was simple to perform, employed inexpensive tools, and was readily accepted by patients, ensuring good patient compliance. None of the patients in the intervention group withdrew from the study due to intolerance, and wearing surgical gloves and elastic socks had no adverse effects on the skin of the hands and feet.

## Discussion

### Effect of gradient pressure therapy on symptoms of paclitaxel-induced peripheral neuropathy

Our results showed a significant difference in the scores and total scores for numbness in the hands and feet, uncomfortable fingers/hands, or toes/feet between the two groups after the fourth cycle of chemotherapy (*P* < 0.05). Additionally, after the sixth cycle of chemotherapy and at the 3-month follow-up, there were significant differences in the scores and total scores for numbness in the hands and feet, uncomfortable fingers/hands, or toes/feet, and cold (*P* < 0.05). This indicates that gradient pressure therapy can effectively prevent or reduce CIPN-related symptoms through a few mechanisms. Firstly, gradient pressure therapy reduces the degree of damage to the microtubule structure [17]. The dynamic balance of microtubules is destroyed, leading to pathological changes in the dorsal root ganglia, and because the peripheral nervous system does not have blood–brain barrier protection, chemotherapy drugs can easily enter and accumulate in sensory neurons. However, axon transport requires the microtubules of the dorsal root ganglion sensory neurons to be maintained, and the hand and foot nerves are the longest axons in the body and the most susceptible to drug effects; therefore, the peripheral nerves are the most common symptomatic sites. Local compression reduces blood flow in peripheral nerve microvessels by stimulating vasoconstriction, thereby reducing the accumulation of chemotherapy drugs in the peripheral nerves and alleviating hand and foot numbness. Secondly, gradient pressure alleviates mitochondrial dysfunction [18, 19]. Taxanes can cause mitochondrial swelling and vacuole formation, thereby impairing mitochondrial function and affecting axon energy supply, leading to sensory neuropathy. Gradient pressure applied to the hands and feet accelerates venous blood and lymphatic fluid reflux. With the increased blood flow velocity, nutrients are transported to the peripheral nerves to a certain extent, thereby reducing impaired mitochondrial axon transport and relieving hand and foot discomfort. Thirdly, chemotherapeutic drugs can directly damage the peripheral nervous system. Damage to large fibers causes abnormal or loss of proprioception, reflex disappearance, and muscle weakness, whereas small fiber damage can cause neuropathic pain and abnormal cold temperature sensations [20]. Applying pressure to the hands and feet reduces pain and sensitivity to cold and warmth by improving local blood circulation. CIPN symptoms worsen with extended chemotherapy cycles and increased cumulative doses; however, most mild and moderate cases gradually improve or disappear after chemotherapy with slow drug metabolism. Severe cases may take longer to recover, consistent with the results outlined by Pachman et al. (2016) [21].

### Effect of gradient pressure therapy on the ADL in patients with taxane-induced peripheral neuropathy

Our results showed that after the fourth and sixth cycles of chemotherapy and at the 3-month follow-up, there were significant differences in the scores for walking, exercise, sleep, sexual activity, chores, enjoyment of life, and the total scores between the two groups (*P* < 0.05). This indicates that gradient pressure therapy can reduce the impact of CIPN symptoms on ADL by preventing or reducing CIPN-related symptoms.

CIPN causes numbness and pain in different parts of the body and to different degrees in patients undergoing chemotherapy, affecting their sleep quality and hindering their performing household chores. In addition, hand numbness affects fine movements, such as button fastening and writing, whereas foot numbness and muscle weakness can cause unstable gait and balance disorders, increasing the risk of falling. Consequently, patients and their families reduce work and exercise for safety reasons. CIPN also causes patients to experience emotional changes, making them unable to enjoy life and reducing their quality of life [22–24].

The impact of CIPN on ADL is related to the severity of CIPN symptoms. As CIPN symptoms increase, their impact on ADL also gradually increases. Gradient pressure therapy administered to the intervention group effectively reduced the CIPN-related symptoms, thereby reducing their impact on daily activities. After chemotherapy, CIPN symptoms gradually ease or disappear, and their impact on ADL gradually decreases.

Scotte´ et al. reported that cryogloves reduce the amount of drugs reaching the nails and skin by causing vasoconstriction, thereby lowering the nail and skin toxicity induced by docetaxel [25]. Eckhoff et al. reported that in a group of 1725 breast cancer patients, the use of cryogloves and long socks was associated with a reduced incidence of docetaxel-induced peripheral neuropathy [26]. Kanbayashi et al. demonstrated that there seems to be no difference in the incidence of paclitaxel albumin-stabilized nanoparticle formulation-induced peripheral neuropathy between cryotherapy and compression therapy [10]. However, cryotherapy has some problems, as some patients feel uncomfortable due to intolerance. Therefore, we can prevent the occurrence of peripheral neuropathy through new feasible methods, and cryotherapy can also be used as an alternative therapy for patients to choose.

This study has some limitations. Conducting research at one hospital may bias specific practices or patient populations at that hospital. The 3-month follow-up period after chemotherapy may not reflect the long-term effects of CIPN and potential late symptoms. The relatively small sample size (80 patients) may limit the generalizability of the findings. Larger studies are needed to confirm these results.

## Conclusions

CIPN symptoms have a significant impact on the daily activities of patients with cancer who are undergoing chemotherapy. Therefore, during treatment, healthcare professionals should promptly identify and assess CIPN symptoms in patients and provide appropriate preventive measures. This study showed that applying a graded pressure intervention in patients receiving white protein paclitaxel chemotherapy could effectively prevent or alleviate CIPN symptoms, reduce the impact of symptoms on patients’ daily activities, and improve their quality of life. None of the patients withdrew because of intolerance, and no compression-related injuries were reported. Thus, this method is suitable for clinical applications. Future research could compare the effectiveness of graded stress interventions with other interventions for the prevention and treatment of CIPN.

## Data Availability

No datasets were generated or analysed during the current study.

## References

[1] Sung H, Ferlay J, Siegel RL, Laversanne M, Soerjomataram I, Jemal A, et al. Global cancer statistics 2020: GLOBOCAN estimates of incidence and mortality worldwide for 36 cancers in 185 countries. CA Cancer J Clin. 2021;71:209–49. 10.3322/caac.21660. 33538338 10.3322/caac.21660

[2] Pearce A, Haas M, Viney R, Pearson SA, Haywood P, Brown C, et al. Incidence and severity of self-reported chemotherapy side effects in routine care: a prospective cohort study. PLoS ONE. 2017;12:e0184360. 10.1371/journal.pone.0184360. 29016607 10.1371/journal.pone.0184360 PMC5634543

[3] Chen CS, Hertz DL. Chemotherapy-induced peripheral neuropathy. Handb Exp Pharmacol. 2023;277:299–337. 10.1007/164_2022_609. 36253554 10.1007/164_2022_609

[4] Burgess J, Ferdousi M, Gosal D, Boon C, Matsumoto K, Marshall A, et al. Chemotherapy-induced peripheral neuropathy: epidemiology, pathomechanisms and treatment. Oncol Ther. 2021;9:385–450. 10.1007/s40487-021-00168-y. 34655433 10.1007/s40487-021-00168-y PMC8593126

[5] Seretny M, Currie GL, Sena ES, Ramnarine S, Grant R, MacLeod MR, et al. Incidence, prevalence, and predictors of chemotherapy-induced peripheral neuropathy: a systematic review and meta-analysis. Pain. 2014;155:2461–70. 10.1016/j.pain.2014.09.020. 25261162 10.1016/j.pain.2014.09.020

[6] Hong JS, Tian J, Wu LH. The influence of chemotherapy-induced neurotoxicity on psychological distress and sleep disturbance in cancer patients. Curr Oncol. 2014;21(4):174–80. 10.3747/co.21.1984. 25089099 10.3747/co.21.1984 PMC4117615

[7] Miaskowski C, Mastick J, Paul SM, Abrams G, Cheung S, Sabes JH, et al. Impact of chemotherapy-induced neurotoxicities on adult cancer survivors’ symptom burden and quality of life. J Cancer Surviv. 2018;12(2):234–45. 10.1007/s11764-017-0662-8. 29159795 10.1007/s11764-017-0662-8 PMC5886787

[8] Tsuyuki S, Senda N, Kanng Y, Yamaguchi A, Yoshibayashi H, Kikawa Y, et al. Evaluation of the effect of compression therapy using surgical gloves on nanoparticle albumin-bound paclitaxel-induced peripheral neuropathy: a phase II multicenter study by the Kamigata Breast Cancer Study Group. Breast Cancer Res Treat. 2016;160:61–7. 10.1007/s10549-016-3977-7. 27620884 10.1007/s10549-016-3977-7

[9] Tsuyuki S, Yamagami K, Yoshibayashi H, Sugie T, Mizuno Y, Tanaka S, et al. Effectiveness and safety of surgical glove compression therapy as a prophylactic method against nanoparticle albumin-bound-paclitaxel-induced peripheral neuropathy. Breast. 2019;47:22–7. 10.1016/j.breast.2019.06.008. 31302389 10.1016/j.breast.2019.06.008

[10] Kanbayashi Y, Sakaguchi K, Ishikawa T, Ouchi Y, Nakatsukasa K, Tabuchi Y, et al. Comparison of the efficacy of cryotherapy and compression therapy for preventing nanoparticle albumin-bound paclitaxel-induced peripheral neuropathy: a prospective self-controlled trial. Breast. 2020;49:219–24. 10.1016/j.breast.2019.12.011. 31901783 10.1016/j.breast.2019.12.011 PMC7375545

[11] Liling W, Shuang C, Hui L. Effect of compression therapy on peripheral neuropathy and psychological distress induced by oxaliplatin in patients with colorectal adenocarcinoma undergoing chemotherapy. Modern Med J China. 2021;23:92–5.

[12] Yamanouchi K, Kuba S, Matsumoto M, Yano H, Morita M, Sakimura C, et al. An evaluation of the efficacy of compression therapy using sleeves and stockings to prevent docetaxel-induced peripheral neuropathy in breast cancer patients. Acta Med Okayama. 2022;76:689–94. 10.18926/AMO/64119. 36549771 10.18926/AMO/64119

[13] YY/T (2011) Pharmaceutical industry standard of the People’s Republic of China: medical anti thrombotic socks. State Food Drug Adm 2011:0851. https://www.chinesestandard.net/Related.aspx/YYT0851-2023 .

[14] National Technical Committee: 35 on rubber and rubber products standardization administration of China (SAC/TC 35) disposable sterilized rubber surgical gloves. GB/T:7543–2020.2020–12–14. https://www.chinesestandard.net/PDF/English.aspx/GBT7543-2020.

[15] Tofthagen CS, McMillan SC, Kip KE. Development and psychometric evaluation of the chemotherapy-induced peripheral neuropathy assessment tool. Cancer Nurs. 2011;34:E10-20. 10.1097/NCC.0b013e31820251de. 21242773 10.1097/NCC.0b013e31820251de

[16] Yu W, Yanni B, Yujie Z, et al. Chinese evaluation tool for chemotherapy-induced peripheral neuropathy and its reliability and validity detection. Nurs J Chinese People’s Liberation Army. 2018;35:14–8 ((in Chinese)).

[17] Ling Z, Jing Z. Research progress on the mechanism of paclitaxel-induced peripheral neuropathy. Chinese J Pain Med. 2022;28:204–9 (in Chinese).

[18] Yuan W, Yan W, Ming M. Mechanism of mitochondrial dysfunction in chemotherapy-induced peripheral neuropathy. Adverse Drug Reactions J. 2019;24:658–63. 10.3760/cma.j.cn114015-20220905-00811.

[19] Junwei W, Xiangqi L. Research progress of peripheral neuropathy induced by taxoid drugs in breast cancer patients. Zhejiang Med J. 2023;45:424–9 (in Chinese).

[20] Ocean AJ, Vahdat LT. Chemotherapy-induced peripheral neuropathy: pathogenesis and emerging therapies. Support Care Cancer. 2004;12:619–25. 10.1007/s00520-004-0657-7. 15258838 10.1007/s00520-004-0657-7

[21] Pachman DR, Qin R, Seisler D, Smith EM, Kaggal S, Novotny P, et al. Comparison of oxaliplatin and paclitaxel-induced neuropathy (Alliance A151505). Support Care Cancer. 2016;24:5059–68. 10.1007/s00520-016-3373-1. 27534963 10.1007/s00520-016-3373-1 PMC5439148

[22] Ying J, Suwen F. Research status and nursing care of peripheral nerve toxicity induced by chemotherapy. Chin J Nurs. 2007;11:995–7.

[23] Tofthagen C. Patient perceptions associated with chemotherapy-induced peripheral neuropathy. Clin J Oncol Nurs. 2010;14:E22–8. 10.1188/10.CJON.E22-E28. 20529785 10.1188/10.CJON.E22-E28

[24] Tofthagen CS, Cheville AL, Loprinzi CL. The physical consequences of chemotherapy-induced peripheral neuropathy. Curr Oncol Rep. 2020;22:50. 10.1007/s11912-020-00903-0. 32323068 10.1007/s11912-020-00903-0

[25] Scotte’ F, Tourani JM, Banu E, et al. Multicenter study of a frozen glove to prevent docetaxel-induced onycholysis and cutaneous toxicity of the hand. J Clin Oncol. 2005;23:4424–9. 15994152 10.1200/JCO.2005.15.651

[26] Eckhoff L, Knoop AS, Jensen MB, et al. Risk of docetaxel-induced peripheral neuropathy among 1725 Danish patients withearly stage breast cancer. Breast Cancer Res Treat. 2013;142:109–18. 24132874 10.1007/s10549-013-2728-2

